# The Influence of Computerized Dynamic Assessment on the Learning Potential of Graphical Analogical Reasoning in Children with Autism: Evidence from Eye-Movement Synchronization

**DOI:** 10.3390/bs15091188

**Published:** 2025-08-30

**Authors:** Kun Zhang, Ying Zhang, Jingying Chen, Yating Dai, Yuanxu Jin

**Affiliations:** 1 National Engineering Research Center for E-Learning, Faculty of Artificial Intelligence in Education, Central China Normal University, Wuhan 430079, China; zhk@mail.ccnu.edu.cn (K.Z.); zhangying_zy@mails.ccnu.edu.cn (Y.Z.);; 2 National Engineering Research Center of Educational Big Data, Faculty of Artificial Intelligence in Education, Central China Normal University, Wuhan 430079, China

**Keywords:** children with ASD, graphical analogical reasoning, computerized dynamic assessment, learning potential

## Abstract

Graphical analogical reasoning ability is crucial for the cognitive development of children with autism spectrum disorder (ASD). However, there are currently no methods available to enhance its analogical reasoning potential. This study aims to explore whether computerized dynamic assessment (CDA) can tap into the potential of children with ASD in graphical analogical reasoning, and simultaneously analyze the influence of the initial abilities of children with autism on their analogical reasoning potential. A total of 71 children with ASD were selected for the study and randomly divided into two groups: (1) the experimental group, namely the computerized dynamic assessment group; (2) the control group, namely the non-computerized dynamic assessment group. Both groups went through three stages: pretest, intervention, and posttest. The research results (including performance, time taken, and eye-tracking analysis) show that compared with non-DA, CDA enables more children to reach medium and high levels of learning potential score. CDA can effectively improve the figural analogical reasoning ability of autistic children in the short term, enhance their attention to learning materials, and boost their answering efficiency. However, this promotion effect is difficult to sustain in the long term. Autistic children with the same initial ability levels show significant differences in learning potential after computerized dynamic assessment because everyone has personalized characteristics. Among children with different initial ability levels, those with lower initial abilities benefit more significantly from dynamic assessment.

## 1. Introduction

Autism spectrum disorder (ASD) is a common and highly heritable neurodevelopmental disorder characterized not only by difficulties in social communication and interaction, sensory abnormalities, and repetitive behaviors but also, more notably, by varying degrees of cognitive impairment (American Psychiatric Association, 2013; DeMyer, 1975). Cognitive skills are essential for children with ASD in terms of scholastic endeavors and everyday existence. Among several higher-order cognitive skills, analogical reasoning is widely recognized as one of the most pivotal. Sternberg proposed the fundamental form of analogical reasoning, represented as A:B::C:D. First, individuals encode objects A and B; then, they consider the relationship between A and B, mapping the relationship between A and C (common attributes); and last, extending this analogy to the correspondence between B and D, generating an appropriate response (Mulholland et al., 1980; Sternberg, 1977). Analogical reasoning involves the ability to understand relationships with similar structures and link them to new relationships to gain knowledge (Stevenson et al., 2013a). It allows individuals to learn from one interaction and apply it to another (Green et al., 2014). This ability enables individuals to effectively evaluate and comprehend the intricate connections and patterns among external information elements, ultimately guiding them toward achieving effective problem-solving solutions (Krulik & Rudnick, 1993). This also enables children to organize and construct new information based on previously acquired knowledge, enhancing their knowledge across various domains (Brown & Kane, 1988; Goswami, 1991). Therefore, analogical reasoning plays a crucial role in children’s developmental processes (Brown & Kane, 1988; Goswami, 1991).

There was a significant correlation between cognitive and analogical reasoning skills. Cognitive dysfunction in children with ASD reduces their ability to understand all of these relationships (Krawczyk et al., 2014). Children with ASD tend to focus on similarities in object features rather than similarities in the relationships between objects (Hetzroni et al., 2019; Krawczyk et al., 2014). Additionally, they often lean toward processing details and trivial information rather than abstract and overall meanings (Aljunied & Frederickson, 2013; Shah & Frith, 1983). Children with ASD often lack the skills necessary to effectively adapt to specific environments (Schopler & Mesibov, 2013). These factors contribute to the difficulty in identifying crucial information in analogical reasoning problems and fully comprehending abstract relational similarities (Green et al., 2014). Consequently, difficulties may arise when there is no apparent literal or surface similarity present (Pexman, 2008).

However, the analogical reasoning abilities of children with ASD may not necessarily be immutable. With proper guidance and support during analogical reasoning tasks, children with ASD are capable of recognizing abstract analogical similarities similar to typically developing (TD) children (Green et al., 2014). For example, virtual reality and other tools can effectively enhance the analogical reasoning abilities of children with ASD by training their social cognition. (Didehbani et al., 2016). However, these studies focused primarily on high-functioning children with ASD (Didehbani et al., 2016), with scant attention given to the broader population of children with ASD. Moreover, most studies have relied on static assessments that capture children’s abilities in the past (Touw et al., 2019), potentially underestimating the cognitive capacities of children with developmental disorders such as autism (Veerbeek & Vogelaar, 2023).

The traditional assessment model separates teaching and assessment, revealing only the learner’s prior knowledge and skills while ignoring their latent abilities. Unlike traditional static assessment methods, dynamic assessment (DA) is believed to capture a person’s learning potential by focusing on abilities that are not yet fully developed (Lidz & Gindis, 2003). It is an assessment method that embeds interventions in the testing procedure (Rashidi & Bahadori Nejad, 2018). DA provides gradual prompts until children are able to complete the task. It focuses on the process of learning rather than the outcome (Lidz & Gindis, 2003). During the assessment process, the mediator endeavors to identify the individual’s potential differences and promptly adjust and improve the teaching. Meanwhile, learners can surpass their actual performance levels with the prompts and guidance of the mediator (Campione & Brown, 1987; Lidz & Gindis, 2003; Rassaei, 2021).

DA avoids the underestimation of cognitive abilities among certain groups of children, such as those with learning difficulties and disadvantaged social situations (Aljunied & Frederickson, 2013; Resing et al., 2020a; Veerbeek & Vogelaar, 2023). Additionally, the practicality of DA in planning interventions is considered one of their important advantages (Grigorenko & Sternberg, 1998; Tzuriel, 1995). It can offer more qualitative and quantitative information about special needs in education (Bosma & Resing, 2012).

Computerized dynamic assessment (CDA) is a type of DA. The emergence of CDA involves interventions through technology devices such as computers. An appealing computer interface can help increase children’s attention and reduce their agitation (Boucenna et al., 2014). Importantly, CDA can provide more detailed records of children’s learning processes and results reports (Poehner, 2008), which can help researchers comprehensively analyse their performance. The use of CDA to help identify and improve the graphic analogical reasoning ability of children with ASD is an optional approach. However, the amount of research on CDA applications involving analogical reasoning for ASD is still limited. Based on existing research, it is unclear whether CDA has a positive impact on analogical reasoning in children with ASD. Therefore, this experiment utilized techniques such as eye-tracking to monitor learners’ eye movement patterns during the learning process to better comprehend the cognitive processes of children with ASD. By integrating performance analysis with eye-tracking data analysis, we aimed to investigate the impact of CDA on children with ASD. The main research questions (RQs) of the study are as follows.

RQ1. Can computerized dynamic assessment unlock the graphical analogical reasoning potential of children with autism spectrum disorder?

RQ2. What is the impact of the initial abilities of children with autism on their analogical reasoning potential?

## 2. Literature Review

### 2.1. Dynamic Assessment

Dynamic assessment stems from Vygotsky’s sociocultural theory (SCT) (Vygotsky & Cole, 1978) and involves embedding intervention measures within testing procedures (Rashidi & Bahadori Nejad, 2018). This assessment method involves three components: the mediator, the intervention (prompt or feedback), and the learner. The mediator presents test items to the learner, who provides feedback. Subsequently, the mediator adjusts the approach based on the feedback and provides learning assistance. DA not only focuses on learners’ current abilities but also emphasizes their potential developmental levels. Depending on the type of prompts used, Lantolf and Poehner categorize DA into interactionist DA and interventionist DA (Lantolf & Poehner, 2008). Interactionist DA involves prompts tailored to learners’ specific situations during interactions between interveners and learners, rendering it more flexible (Lantolf & Poehner, 2008). While interactionist assessment is finer-grained, it is also more time- and labor-intensive (Ahmadi & Besharati, 2017; Lantolf & Poehner, 2011). Interventionist DA employs predesigned prompts to intervene with learners, gradually presenting prompts according to the intervention plan. Given the utilization of computer technology in this large-scale intervention study, interventionist DA was adopted in this study. Based on different intervention processes, Sternberg and Grigorenko categorized DA into cake format DA and sandwich format DA (Sternberg & Grigorenko, 2001, 2002). Cake format DA combines assessment and intervention, providing intervention support to learners during assessment until they answer correctly. The sandwich format DA separates the two phases, consisting of pretest, intervention, and posttest phases. This study employed the sandwich DA format to better observe the differences in performance of children with ASD across three stages.

Several studies have focused on children with intellectual disabilities and learning difficulties utilizing DA. Fabio’s research showed that, compared to static assessments, DA could provide a comprehensive assessment of the intellectual level of children with attention deficit hyperactivity disorder. It could provide more detailed information and more accurate predictions of learning ability (Fabio et al., 2022). Aljunied’s study revealed significant improvements in learning outcomes for children with ASD after receiving DA, regardless of their level of performance intelligence quotient (Aljunied & Frederickson, 2013). The researcher emphasized the consistent findings of enhanced learning outcomes through DA for children with ASD, as well as for children with Down syndrome and other learning difficulties (Aljunied & Frederickson, 2013). These findings suggest that DA has the potential to enhance the learning abilities of children with developmental disorders.

### 2.2. Computerized Dynamic Assessment for Graphical Analogical Reasoning

Computerized dynamic assessment (CDA) is a form of dynamic assessment. It typically utilizes devices such as smartphones (Rassaei, 2021), computers (Vogelaar et al., 2021), robots (Resing et al., 2020b), 3D virtual environments (Passig et al., 2016), and virtual reality (Didehbani et al., 2016) to provide generalized interventions for learners. Its core is to use computer technology to provide learners with hints and other assistance, and to capture learners’ performance in learning tasks, thereby stimulating their learning potential (Resing & Elliott, 2011). Numerous studies have shown the effectiveness of CDA in enhancing learning outcomes. For example, Resing’s research demonstrated that CDA can improve children’s inductive reasoning abilities and analytical skills in problem solving (Resing et al., 2012). Vogelaar’s study also indicated that children trained with CDA show greater improvement in analogical reasoning than untrained children do. Importantly, CDA can reduce the impact of executive function (EF) on children (Vogelaar et al., 2021). Extensively defined, EF is characterized as the comprehensive control of cognitive processes that are directed toward goals and oriented toward the future (Stuss, 2011). This is a particularly important part of the context of research on children with ASD, as their executive functions are generally impaired and serve as a limiting factor in their cognitive development (Demetriou et al., 2019).

Most of these studies have focused on TD children. However, children with ASD differ from these children in terms of intelligence (Aljunied & Frederickson, 2013). There is no evidence to suggest that children with ASD benefit from the same intervention as other children. Moreover, few studies have focused on exploring children’s cognitive pattens during the learning process. For example, correlations between time spent answering questions and learning outcomes and correlations between various indicators of learning outcomes were identified. Furthermore, research currently provides limited descriptions of children’s learning outcomes, which are often evaluated solely through test scores without consideration of changes in their attention. Therefore, further research is needed to investigate the reliability of CDA for identifying Children with ASD and the effects of CDA on learning outcomes (Aljunied & Frederickson, 2015).

### 2.3. Learning Potential

The idea of assessing learning potential has been around for a long time; in 1924, the well-known researcher Thorndike defined intelligence as “the ability to learn”, followed by Vygotsky’s concept of the zone of proximal development (ZPD). He defined it as the gap between the level of problem solving that can be achieved with adult assistance in guided situations and the level of problem solving that can be achieved in independent activities. According to Vygotsky, the key is not to stop the process of development that has already been accomplished, but to “identify the dynamics of the child’s development” to promote it (Allal & Ducrey, 2000). The introduction of this concept emphasized the feasibility and significance of promoting children’s intellectual development in teaching and education and gave further impetus to the research on the assessment of learning potential.

Based on these studies, scholar Luria gave methods and recommendations for assessing learning potential. He suggested that children who originally scored low on intelligence tests could be re-diagnosed (Luria, 1961). If, after the same length of training, some of the children showed a significant increase in intelligence while others showed no improvement, then the latter were intellectually disabled (Budoff & Friedman, 1964). This series of studies has important implications for understanding and defining learning potential, which can be regarded as the potential degree of enhancement of an individual’s abilities that may be achieved under specific guidance and training conditions, as opposed to the abilities that have already been demonstrated the present time, and focuses more on the possibilities of the individual’s future development under appropriate environments and guidance (Sternberg & Grigorenko, 2002).

The calculation of the learning potential score (LPS) was based on the equation proposed by Kozulin and Garb. The calculation of LPS is based on the pre- and post-test scores. The scoring criteria for LPS were as follows: LPS ≥ 1.0 was classified as high, 1.0 > LPS ≥ 0.71 was classified as medium, and LPS < 0.71 was classified as low. The equation is as follows (Kozulin & Garb, 2002):

(1) $$LPS=\frac{\left(S_{post}-S_{pre}\right)}{S_{max}}+\frac{S_{post}}{S_{max}}=\frac{2S_{post}-S_{pre}}{S_{max}}$$

*S_pre_* and *S_post_* are pre- and posttest scores, respectively, and *S_max_* is the maximal obtainable score.

(2) $$LPS=\frac{2S_{mediated}-S_{actual}}{S_{max}}$$

Lantolf et al. introduced the concept of mediated scores based on the formula proposed by Kozulin (Poehner & Lantolf, 2013). Scholar Zhijun Sun pointed out that Lantolf’s formula cannot accurately reflect the learning potential defined by Vygotsky. Then, in 2023, Zhijun Sun sorted out the LPS formula and proposed a new one based on previous studies (Calculated based on the mediated score), as shown in Equation (2). “*S_mediated_*” is the mediated score, which refers to the test score obtained after giving the prompt. “*S_actual_*” is the actual score, which refers to the score obtained on the first attempt in the intervention phase without any prompting. *S_max_* is the maximum score that can be obtained in one test. Through the mediated score (*S_mediated_*) and actual score (*S_actual_*), the impact of the intervention on the subjects’ learning potential can be measured more precisely, reflecting the subjects’ ability to improve under the condition of having external support. Sun et al. conducted empirical research verification, proving the rationality of the formula (Izadi et al., 2023; Mehri Kamrood et al., 2021; Sun et al., 2023). This method was adopted in the experimental group of this study.

It’s important to note that the LPS formula was proposed to calculate the potential abilities of learners under dynamic assessment. In this study, calculating the LPS for the control group without CDA implementation (No mediated score) is mainly for comparison with the experimental group to show the effect of CDA. Therefore, the most commonly used formula proposed by Kozulin (without mediation scores) was selected for calculating the LPS of the control group.

## 3. Materials and Methods

### 3.1. Participants

To ensure that the experimental design of this study was sufficient, G*power 3.1 software (University of Düsseldorf, Düsseldorf, Germany) (Faul et al., 2007) was used to estimate the required sample size. An effect size of 0.25 and an alpha of 0.05 were used. The analysis showed that a total of at least 44 participants were needed to achieve a statistical power of 0.95.

This study was conducted at a special education institution in a certain city in Hubei Province, China. All children have experience using electronic devices such as tablets in their daily study or life, which provides the necessary basic conditions for this research. In addition, all participants were required to meet the following criteria: (1) meet the criteria for Autism Spectrum Disorder (ASD) in the Diagnostic and Statistical Manual of Mental Disorders, 5th Edition (DSM-5); (2) be diagnosed with autism by a top-level (Grade III Class A or above) professional hospital; (3) have no history of other mental illnesses or brain injuries; (4) have normal vision; (5) have flexible fingers; (6) be able to understand basic instructions; (7) have not participated in similar experiments and have never seen the materials of this experiment before.

This study recruited 71 children with autism from the institution. Five children did not participate in the posttest, so they were excluded from the data analysis, resulting in a total of 66 Children with ASD (51 boys, accounting for 77%, and 15 girls, accounting for 23%). The ratio of male to female autistic individuals ranges from 2:1 to 5:1 (Lord et al., 2020), and the sex ratio of participants in this study is consistent with this report. The average age of the 66 participants was 5.80 years (SD: 1.39, min: 3.00, max: 9.00). This study adopted a single-factor pre- and post-test design. Participants were randomly assigned to two groups, namely the CDA group and the Non-CDA (NDA) group. The CDA group received computerized dynamic assessment, while the NDA group served as the control group, only completing the corresponding tests without any intervention or assistance. The learning and testing contents of the two groups were exactly the same. A Mann–Whitney U test revealed no significant differences in age between the two groups (z = −1.897, *p* = 0.058). There was also no statistical difference in the prior knowledge abilities between the two groups of children (see the specific data in Section 4.1 and Section 4.2). Basic demographic information is presented in Table 1.

Informed consent was obtained from the special education agency and parents prior to the start of the experiment. Our research team signed a confidentiality agreement with the special education agency. The study was approved and supported by the institutional review board.

### 3.2. Learning Materials

This study adapted the research materials from Whitely’s study (Whitely & Schneider, 1981) and established eight transformation rules: quantity, size, color, shape, position (nesting, overlapping), and transformation (inversion, rotation). Each rule was accompanied by a corresponding question, as outlined in Table 2.

The experiment was divided into a total of three phases, i.e., pretest–intervention–posttest. Each phase consisted of eight questions, and the eight questions followed the transformation rules described above. The content of the questions in the pretest and intervention phases was identical, while the questions in the posttest phase were isomorphic to the questions in the first two phases.

**Figure 1:**
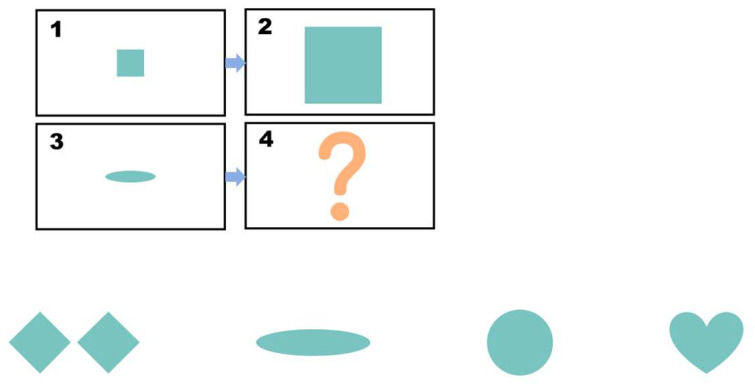
Pretest: The four boxes above represent the questions, and the four figures below are the options. Participants need to apply the transformation rule from box 1 to box 2 to the change from box 3 to box 4, and then infer the content of box 4. In this study, all the questions are presented in this layout.

Pretest. This assessment consists of eight items designed to evaluate children’s prior knowledge of graphic analogical reasoning. Each item includes four options, with only one being the correct answer, as shown in Figure 1. A correct answer is awarded four points, and incorrect answers receive no points. The total pretest score is calculated by adding the scores of all correct answers, with a maximum score of 32. The Cronbach’s alpha coefficient of the test was 0.770. Participants were asked to complete this section of the test within 5 min.

**Figure 2:**
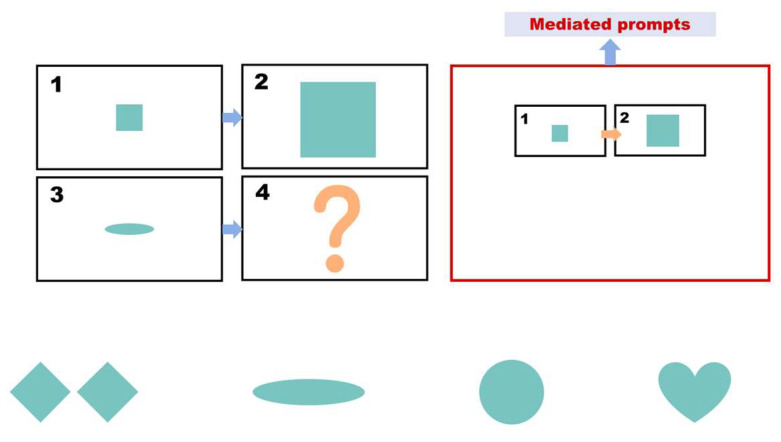
Intervention.

During the intervention phase, the same set of questions used in the pre-test phase is employed. However, for each incorrect answer, participants receive a prompt (as shown in Figure 2). The prompts are divided into three levels, with each level lasting approximately 20 s. Table 3 presents the prompt design and scoring rules used. If a child answers incorrectly after receiving prompts from all three levels, the question is scored as zero, and the participant proceeds to the next question. The prompts are presented in the form of animated videos. Participants are required to complete the test within 10 min during this phase.

**Figure 3:**
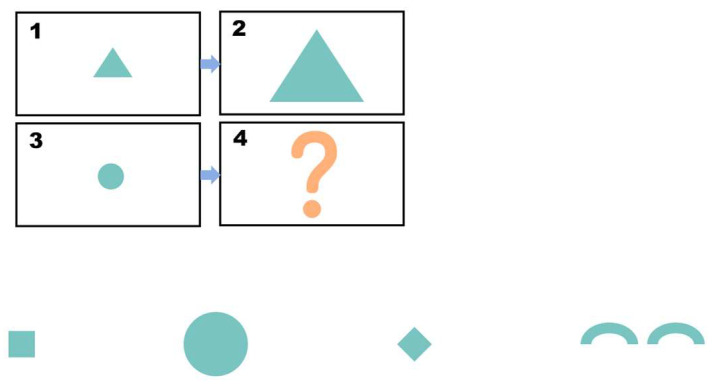
Posttest.

Posttest. The posttest consists of 8 questions. The questions are like the pretest questions, as shown in Figure 3. Each correct answer is worth 4 points, and each incorrect answer is worth 0 points. No hints are provided, and the maximum score is 32 points. The Cronbach’s alpha coefficient for the post-test is 0.759. Participants are required to complete this part of the test within 5 min.

### 3.3. Hardware and Software Equipment

The experimental materials were designed with user-friendliness in mind to prevent fatigue among children with autism during the intervention process (Zhang et al., 2017). The experimental materials were presented to participants on an all-in-one machine with a screen resolution of 1920 × 1080 pixels. The touchscreen capability of an all-in-one machine enhances participants’ engagement and attention (Foshay & Ludlow, 2005).

**Figure 4:**
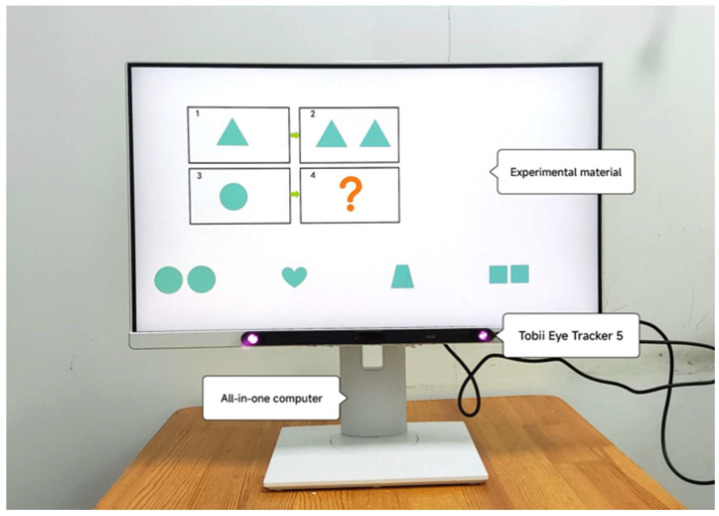
Experimental equipment.

**Figure 5:**
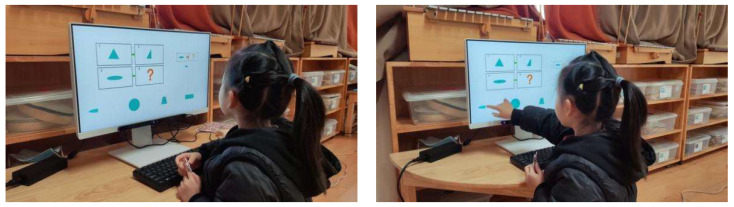
Experimental implementation environment.

Eye tracking is a sensing technology that utilizes a camera and near-infrared light source to track real-time eye movement through certain algorithms. In this study, eye movement data were collected using the Tobii Eye Tracker 5 (Tobii, Stockholm, Sweden) desktop eye tracker. The eye tracker was positioned beneath the all-in-one machine screen, connected to the machine, and had a viewing angle of 40 × 40 degrees as per the experimental setup shown in Figure 4 and on-site experimentation in Figure 5. We divided the areas of interest into a question section, an answer section, and a mediated prompt section. The eye-tracking data were imported into OGAMA 5.1 (open-source software) to define regions of interest, filter participant data, and generate eye-tracking heatmaps and trajectories.

### 3.4. Experimental Design

**Figure 6:**
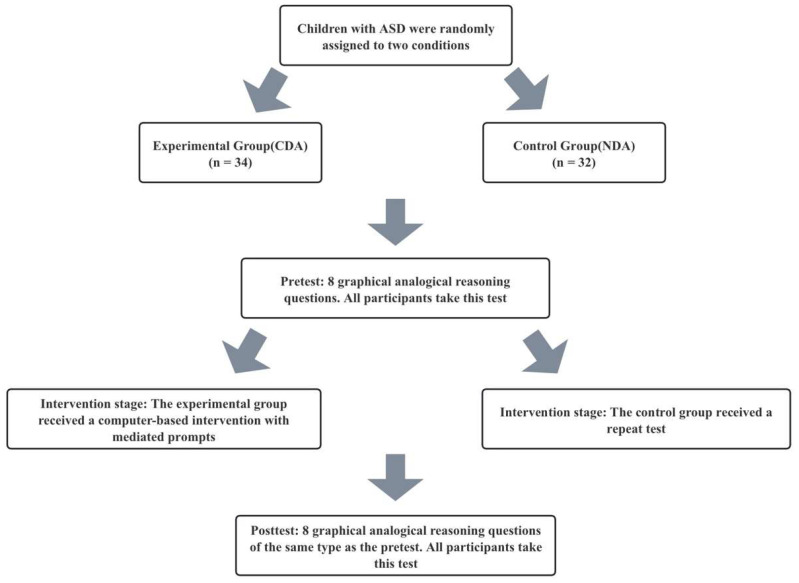
Experimental flowchart.

This study was conducted in a well-lit classroom where children sat in front of all-in-one machines and other devices. Prior to the start of the experiment, demographic questions were answered by the parents or teachers of the participants. Subsequently, the experimenter introduced the experiment and explained the rules of the analogy reasoning test to the children. Under the guidance of the experimenter, the participants performed eye-tracking calibration using Tobii Eye Tracker 5 to adjust the distance between themselves and the computer display for optimal eye-tracking data recording. Once calibration was completed, both groups of participants independently completed the same pretest. Later, in the “CDA” group, participants first independently selected their answer choices, advancing to the next question if the response was correct. If the answer was incorrect, the participants received three levels of prompt intervention. In the “NDA” group, participants underwent testing with the same questions as those in the “CDA” group but without any prompts. Finally, both the “CDA” and “NDA” groups underwent the same posttest. The experimental procedure for each child lasted approximately 20 min. The flowchart of the experiment is shown in Figure 6.

This study adheres to the principles of Universal Design for Learning (UDL) by incorporating computerized technologies, such as dynamic assessment platforms and eye-tracking systems (Rose, 2000). During the intervention stage, 2D animations with synchronized sound and images are used as an intervention measure to support learners with visual and hearing impairments. Additionally, non-invasive data collection captures eye movement patterns. This allows learners with diverse expressive capabilities to demonstrate their understanding through alternative modalities. The CDA framework incorporates multi-level prompts, and the intervention’s prompt hierarchy is presented dynamically based on the learner’s mediated score, constructing personalized learning pathways. Thus, this systematic technological integration transforms CDA into an instrument of inclusive pedagogy.

### 3.5. Data Analysis

In the data analysis process, this study excluded samples with missing data for any of the three tests and included only complete sample data. Due to the special characteristics of children with ASD, they typically exhibit symptoms such as hyperactivity and lack of focus, resulting in suboptimal eye-tracking data. Many children were unable to remain seated in front of the computer continuously or maintain an appropriate eye-tracking distance, resulting in incomplete eye-tracking data recording and less time recording gaze duration compared to their actual test duration. During the eye-tracking data analysis, 23 participants were excluded, resulting in a final sample size of 43 participants.

This study first conducted a Shapiro–Wilk normality test on the test scores, response times, and various eye-tracking data. Many of the data did not meet the requirements for a normal distribution (*p* < 0.05). Therefore, to analyse these data, a generalized estimating equation (GEE) approach was used, and the median and quartiles were used to describe the distribution characteristics. During this process, DA (CDA, NDA) was used as a between-subject factor, and the testing phase (pretest, intervention, posttest) was used as a within-subject factor. GEE analyses were also conducted on learning outcomes and eye-tracking measures, focusing on main and interaction effects, as well as simple effects, to measure the impact of CDA on participants’ analogy reasoning ability and gaze patterns. The significance level was set to 0.05, and Bonferroni corrections were used.

Data analysis and visualization were carried out using SPSS 29.0.1, Ogama 5.1, G*Power 3.1, Origin 2021, and GraphPad Prism 10.0.2 for this study.

## 4. Results

### 4.1. Analysis of Test Scores

**Figure 7:**
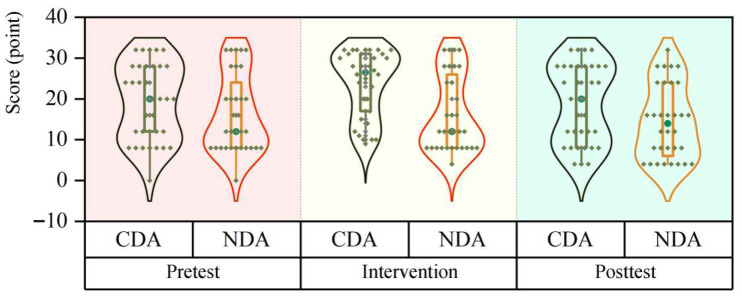
Changes in two sets of scores at three stages.

GEE analyses revealed a significant main effect of group (CDA group and NDA group) on learners’ test scores (Wald $\chi^{2}$ = 5.539, *p* = 0.019), a significant main effect of phase (pretest–intervention–posttest phase) on test scores (Wald $\chi^{2}$ = 30.886, *p* < 0.001), and a highly significant interaction of group and phase (Wald $\chi^{2}$ = 15.711, *p* < 0.001). The changes in the scores of the two groups are presented in violin plots showing the median, upper quartile and lower quartile of the data, as well as the central distribution of all data, as shown in Figure 7.

Simple between-group effect analyses (Mann–Whitney U test) revealed no significant differences between the two groups at both pretest (z = −1.213, *p* = 0.225) and posttest (z = −1.900, *p* = 0.057). However, the CDA group performed significantly better than the NDA group during the intervention phase (z = −3.214, *p* = 0.001).

The results of the within group simple effects analysis (Friedman’s test) showed that there was no significant difference between the test scores of the NDA group in the three different phases (pretest, intervention prompt and posttest) ($\chi^{2}$ = 5.614, *p* = 0.060). However, there was a significant difference in scores across the three phases for the CDA group ($\chi^{2}$ = 16.467, *p* < 0.001). A Wilcoxon signed rank test was performed to determine which phases were significantly different. The results showed that scores at the intervention prompt stage were significantly higher than at the pretest stage (z = −3.775, *p* < 0.001), but scores at the posttest stage were significantly lower than scores at the intervention prompt stage (z = −4.002, *p* < 0.001). There was no significant difference between posttest and pretest scores (z = −0.548, *p* = 0.584). Specific results are presented in Table 4.

### 4.2. Time Use Analysis

**Figure 8:**
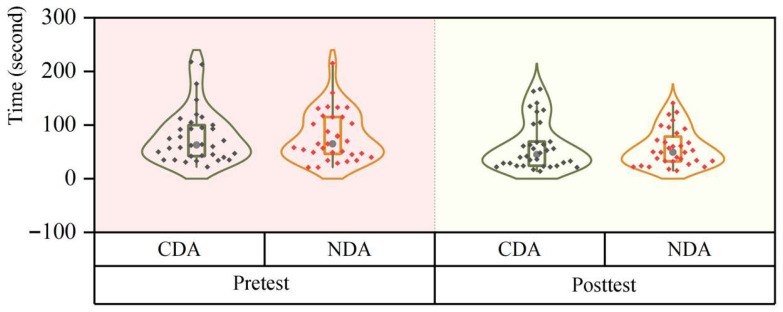
Changes in time spent by the two groups in the three phases.

The GEE results showed that there was no significant main effect of group (Wald $\chi^{2}$ = 0.887, *p* = 0.346) and stage (Wald $\chi^{2}$ = 0.032, *p* = 0.858) on the time learners took to answer the questions, and there was no significant interaction between the two (Wald $\chi^{2}$ = 0.961, *p* = 0.327), as shown in Figure 8 (in seconds).

Results of between-group simple effects (Mann–Whitney U test) showed no significant difference in answer times between the CDA and NDA groups at both the pretest (z = 0.160, *p* = 0.873) and posttest (z = −0.154, *p* = 0.878) stages.

Within-group simple effects analyses (Wilcoxon signed-rank test) revealed that in the CDA group, there was a significant difference in answer times between the two phases (z = −2.078, *p* = 0.038), with a significant reduction in posttest time compared to pretest time. In the NDA group, there was also a significant difference in the answer times between the two phases (z = −3.498, *p* < 0.001), and the answer times in the posttest phase were similarly significantly shorter than in the pretest phase. The results of the time spent analysis are in Table 5.

### 4.3. Learning Potential Analysis

**Figure 9:**
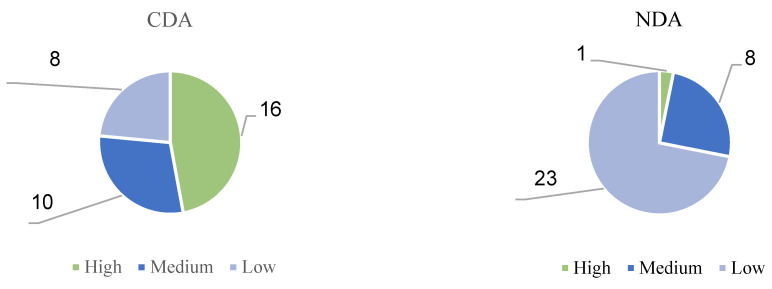
CDA and NDA groups, percentages of people with different LPS levels.

As shown in Figure 9, in the CDA group, 16 children, or 47 percent of the group, had high LPS levels. In addition, 10 children (29%) achieved a moderate LPS level. The proportion of children with low LPS levels was relatively low at 24%. In the NDA group, only one child had a high LPS level, and eight children had a moderate LPS level. It is noteworthy that the number of children with low LPS levels in the NDA group was quite high, with a total of 23 children, or 72% of the total number of children in the experiment.

**Figure 10:**
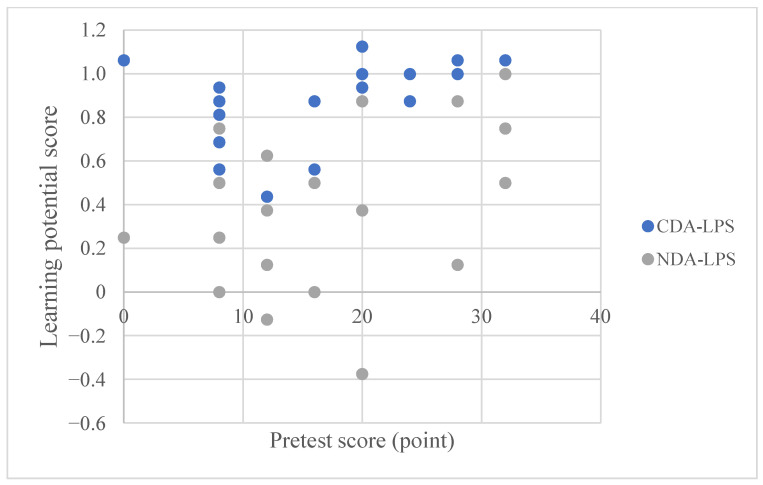
Correspondence between the pretest scores of children in the CDA and NDA groups and their LPS scores.

Figure 10 shows the relationship between the pretest scores and the LPS for the two groups of children. The graph shows that when the children’s pretest scores were the same, their LPS scores differed. Specifically, for children with similar prior knowledge levels, their learning potential is different, which shows that each autistic child has individual characteristics. CDA can stimulate children’s learning potential and help children reach a higher level of learning potential (Tudge, 1992; Vygotsky & Cole, 1978).

### 4.4. Fixation Count Analysis

GEE analyses revealed no significant main effect between groups (Wald $\chi^{2}$ = 3.454, *p* = 0.063), a significant main effect between stages (Wald $\chi^{2}$ = 6.938, *p* = 0.031), and a non-significant interaction between group and stage in terms of the number of gaze points (Wald $\chi^{2}$ = 3.180, *p* = 0.204), as shown in Table 6 shows.

Between-group comparisons using the Mann–Whitney U test indicated that there was no significant difference between the two groups at the pretest (z = −0.139, *p* = 0.890) or posttest (z = −1.096, *p* = 0.273) stages. Notably, a significant difference was observed between the two groups during the intervention phase (z = −2.116, *p* = 0.034), in which the CDA group had a significantly higher number of gazes than the control group.

Within-group analyses using Friedman’s test indicated that there was no significant difference between the number of gazes across the three phases for either the CDA group ($\chi^{2}$ = 2.154, *p* = 0.341) or the NDA group ($\chi^{2}$ = 0.651, *p* = 0.722).

### 4.5. Fixation Duration Mean Analysis

According to the GEE analysis, there were no significant main effects of group (Wald $\chi^{2}$ = 0.954, *p* = 0.329) or phase (Wald $\chi^{2}$ = 2.694, *p* = 0.260) on fixation duration mean. Additionally, there was no significant interaction effect between group and phase (Wald $\chi^{2}$ = 1.448, *p* = 0.485), as presented in Table 7 (unit in milliseconds).

Further analysis using the Mann–Whitney U test revealed no significant differences among the three phases for group comparisons.

Similarly, within-group analysis using the Friedman test revealed no significant differences in gaze time across the three phases for either the CDA group ($\chi^{2}$ = 0.316, *p* = 0.854) or the NDA group ($\chi^{2}$ = 1.000, *p* = 0.607). Pairwise comparisons between the three phases in each group also indicated no significant differences between the CDA and NDA groups.

### 4.6. Eye Movement Hotspot Map

Table 8 presents the results of the eye-tracking analysis using Ogama software. The data show that in all experimental phases, the CDA group and the NDA group had significantly higher gaze concentration in regions C and D than in regions A and B. (Analogical Reasoning topic form A:B::C:D) It is noteworthy that the mediating cue that appeared in the CDA group greatly attracted the participants’ attention.

### 4.7. Analysis of the Number of Transitions Between Areas of Interest

**Figure 11:**
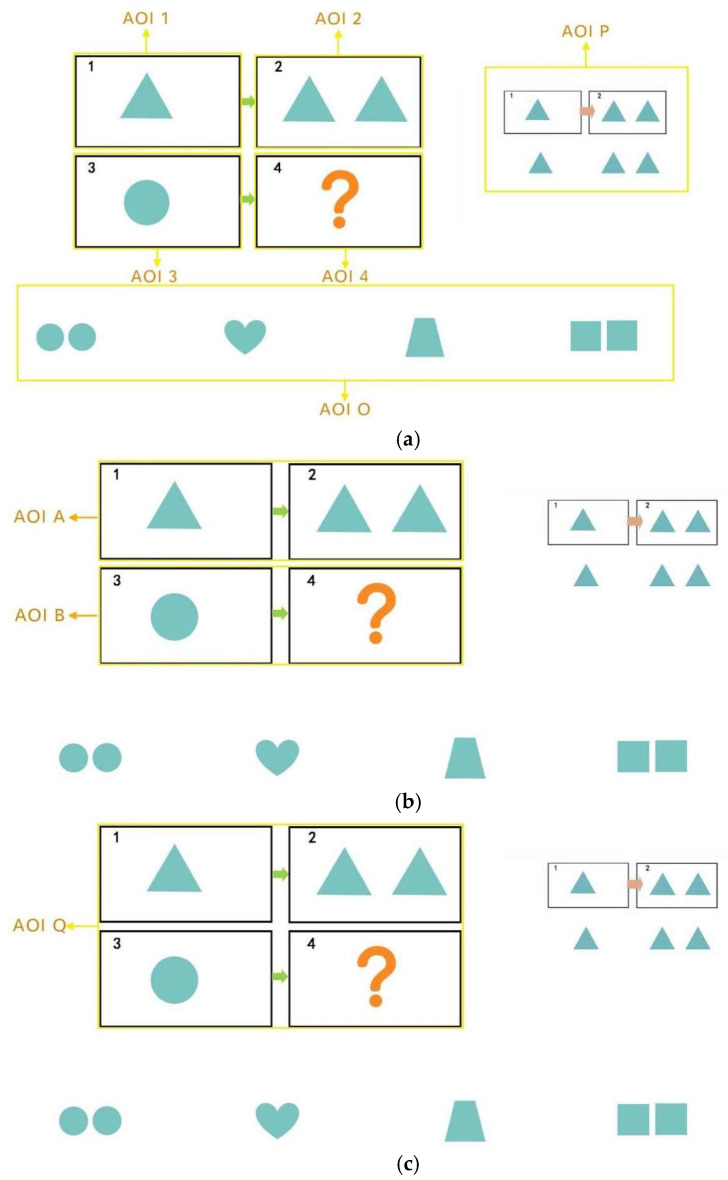
(**a**) Delineation of areas of interest. (**b**) Delineation of areas of interest. (**c**) Delineation of areas of interest.

In this study, the question items A, B, C, and D (A:B::C:D) were divided into four Areas of Interest (AOI), i.e., A: AOI 1, B: AOI 2, C: AOI 3, and D: AOI 4 by combining AB into AOI A, and CD into AOI B. We designated the entire group of question items as AOI Q, the response options as AOI O, and the prompt section as AOI P, as shown in Figure 11.

**Figure 12:**
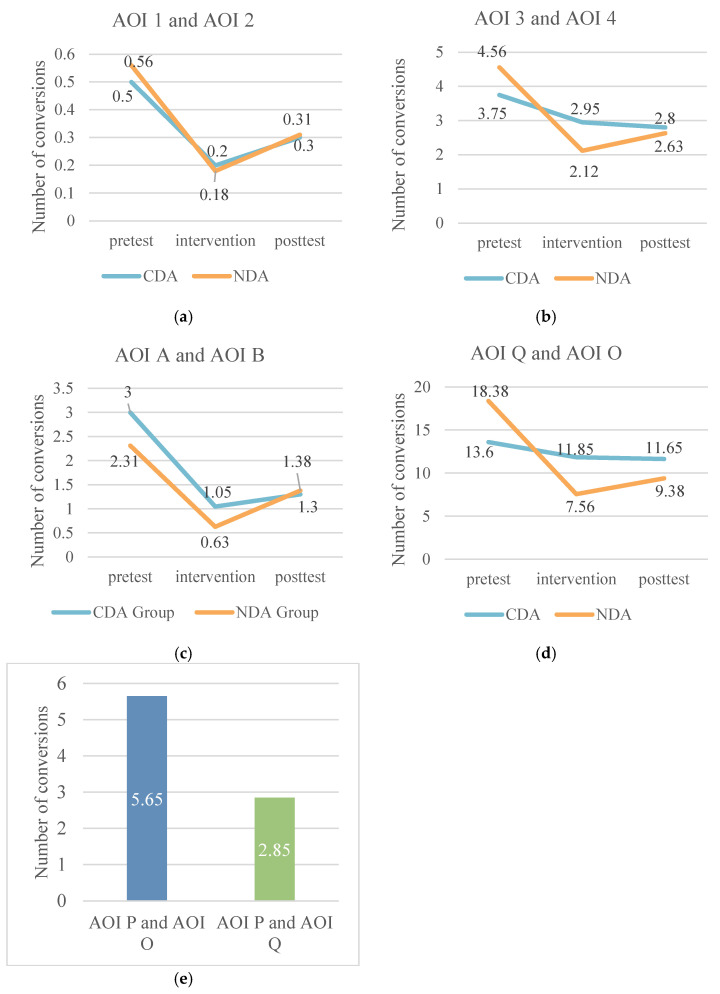
(**a**) Statistics on the number of conversions between areas of interest 1 and 2. (**b**) Statistics on the number of conversions between areas of interest 3 and 4. (**c**) Statistics on the number of conversions between areas of interest A and B. (**d**) Statistics on the number of conversions between areas of interest Q and O. (**e**) Statistics on the number of transitions between area of interest P and O and Q.

The study analyzed the number of area-of-interest transitions between the control and experimental groups, using the number of gaze transitions per subject per capita normalized by the number of people for comparison between groups. Overall, the number of gaze transitions between AOIs 3 and 4 was generally higher than that between AOIs 1 and 2, and the number of transitions between Q and O was significantly higher than that between A and B. Specifically, the number of gaze transitions decreased in all groups at the intervention stage compared to the pretest stage, with the largest decreases occurring between AOIs 1-2 and A-B. Except for the CDA group, the number of transitions between AOIs 3 and 4 and Q and O showed a slight decrease at the posttest stage, and the remaining groups showed a slight decrease in the number of transitions between AOIs 3 and 4. The rest of the groups showed a rebound trend in the posttest stage, except for the CDA group in the posttest of AOI 3-4 and Q-O, where the number of transitions still decreased slightly. Notably, the CDA group had a significantly higher number of gaze transitions to P-O than to P-Q during the intervention phase. Specifically, as shown in Figure 12.

### 4.8. Correlation Analysis of Time and Test Scores

This study adopted the calculation method used by scholar Izadi in his study to determine the actual score, mediator score and gain score (Izadi et al., 2023). Where gain score is the difference between mediator score and actual score.

**Figure 13:**
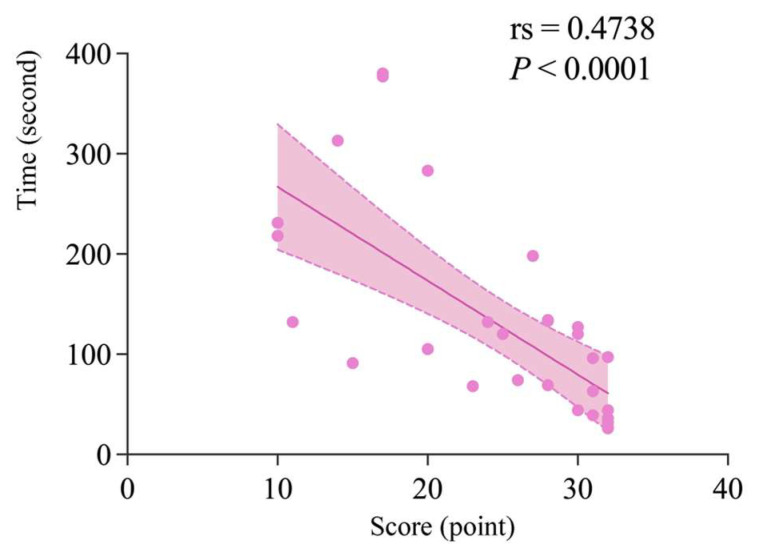
Correlation analysis (including 95% confidence intervals) between time in intervention phase and scores for CDA group.

**Figure 14:**
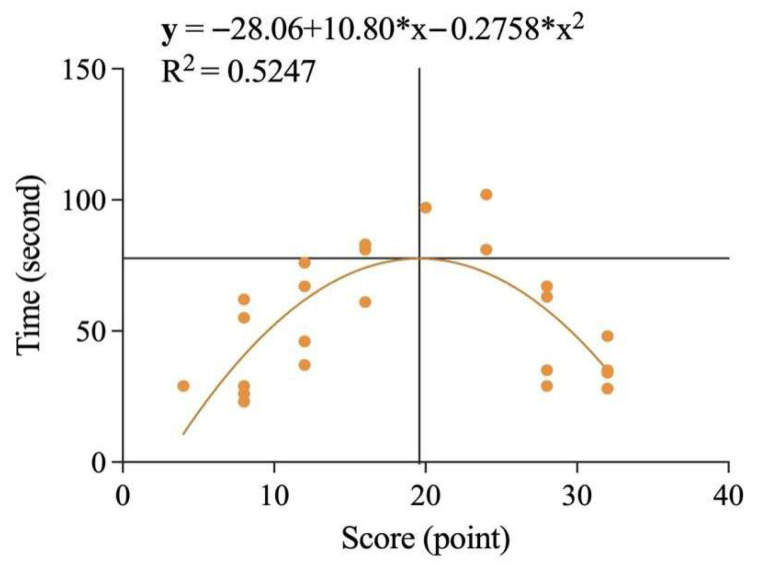
Correlation analysis between time and score in the intervention phase in the NDA group.

After the Shapiro–Wilk test revealed that the data did not conform to a normal distribution, this study conducted Spearman’s correlation analysis between the time spent and the scores of the two groups of students at the pretest, intervention, and posttest stages. The results showed that there was no significant correlation (*p* > 0.05) between time spent and scores for either the CDA or NDA groups, either at the pretest or posttest stage. However, during the intervention phase, there was a significant correlation between scores and response times for both groups. As shown in Figure 13, in the CDA group, there was a significant negative correlation between score and time spent (rs = 0.4738, *p* < 0.0001), indicating that the higher the score, the shorter the reaction time and vice versa. For the NDA group, although direct analysis showed no significant correlation between scores and response times, we found when plotting the data, as shown in Figure 14, that the fit was good after removing outliers and applying a second-order polynomial fit (y = −28.06 + 10.80*x − 0.2758*x2, R2 = 0.5247). Based on this figure, it was found that there was a positive correlation between score and reaction time when children’s scores were below 19.58 out of 32, and a negative correlation between score and reaction time when children’s scores were above 19.58.

### 4.9. Correlation Analysis Between Actual Scores and Mediator Scores

**Figure 15:**
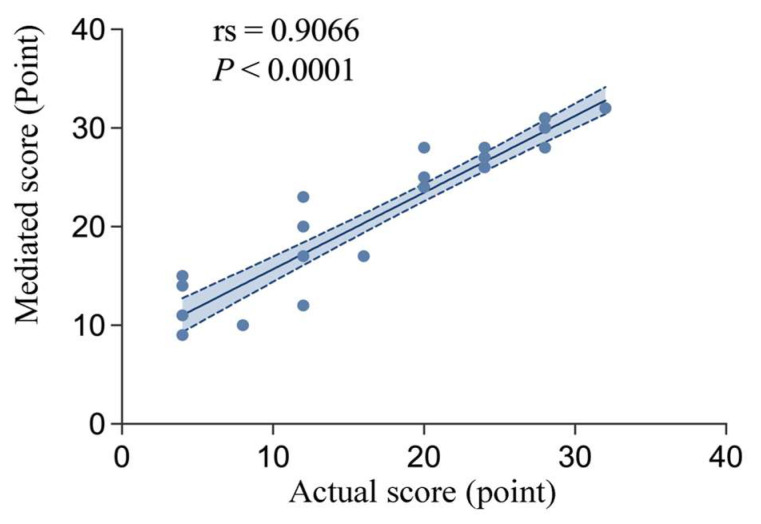
Correlation analysis between the actual and mediated scores.

A Spearman correlation analysis was conducted to examine the relationship between actual scores and mediation scores, and the results showed a significant positive correlation between the two (rs = 0.9066, p < 0.0001). As shown in Figure 15, the higher the actual score, the higher the mediation score.

### 4.10. Correlation Analysis of Actual Scores and Gain Scores

**Figure 16:**
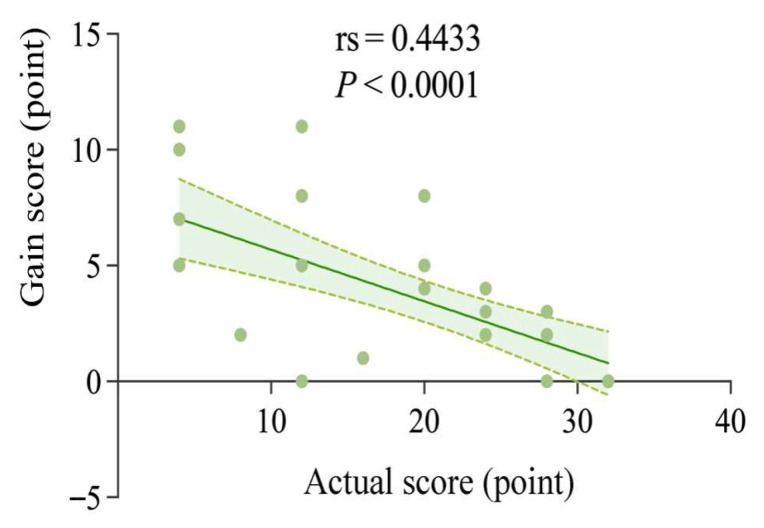
Correlation analysis between the actual score and gain score.

Spearman’s correlation analysis of the relationship between the actual and mediator scores showed a significant negative correlation (rs = 0.4433, p < 0.0001). As shown in Figure 16, the higher the actual score, the lower the gain score; conversely, the higher the gain score, the lower the actual score.

## 5. Discussion

### 5.1. Discussion of the Impact of Computerized Dynamic Assessment

Analysis of the results of the graphical analogical reasoning scores and other outcomes of children with autism showed that computerized dynamic assessment was effective in promoting the development of their graphical analogical reasoning ability. Compared with the non-dynamic assessment group (NDA group), more children in the computerized dynamic assessment group (CDA group) reached the intermediate and advanced learning potential levels. During the intervention phase, with the help of mediated prompts, children with autism in the CDA group performed significantly better than those in the NDA group who did not receive prompts. Analysis of the eye-tracking data revealed the reason for the difference in performance between the two groups: during the intervention phase, children in the CDA group gazed at significantly more points than those in the NDA group. Meanwhile, the CDA group had frequent transitions between the mediated cue area and the question and option areas due to the presence of the mediated cue, suggesting that the mediated cue effectively attracted the children’s attention, and that the increased attention helped to promote children’s thinking, which in turn led to successful completion of the analogical reasoning task (Vogelaar et al., 2021). These findings are consistent with those of previous studies of typically developing children (Resing et al., 2020b; Vogelaar et al., 2021), suggesting that computerized dynamic assessment is not only effective for typically developing children, but also reduces the impact of executive functioning on children with autism (Vogelaar et al., 2021), giving them the ability to break through to higher levels (Bakhoda & Shabani, 2019), which in turn produces favorable results. Children with autism often have varying degrees of deficits in executive functioning, which includes a variety of aspects such as cognitive flexibility, working memory, and inhibitory control, and these deficits severely limit their ability to learn and live. Computerized dynamic assessment, with its unique interactive, adaptive, and personalized features, can give prompting support to autistic children based on their real-time performance, thus effectively avoiding assessment bias that may be caused by executive function deficits.

Further analysis of the posttest data shows that CDA only enhances the graphical analogical reasoning ability of children with autism in the short term, but the impact is difficult to sustain, with a decreasing trend in their posttest scores, which are not significantly different from the pretest. From the hotspot map, it can be visualized that children pay more attention to the interest areas C and D, often neglecting to analyze A and B. Moreover, children made significantly more transitions between regions of interest C and D than between A and B. The number of transitions between regions of interest decreased significantly in the intervention, although there was a slight rebound at the posttest. This suggests that children with autism have difficulty sustaining attention on specific objects, lack depth of cognitive engagement, lack further reasoning, and are prone to incorrect answers (Thibaut & French, 2016). Previous studies have shown that not all learners benefit from CDA due to the fact that everyday learning does not sufficiently foster divergent thinking in children with autism (Zbainos & Tziona, 2019).Aljunied noted that children with autism in special schools have less exposure to learning abstract analogical problems compared to children in normal schools (Aljunied & Frederickson, 2013; Aljunied & Frederickson, 2015). In addition, the limited time available for assessment is an important limiting factor. Therefore, time is a key factor to be emphasized in subsequent analogical reasoning interventions for children with autism. Several scholars have suggested that larger sample sizes and longer intervention times are needed to achieve significant results (Ahmadi & Besharati, 2017; Hung et al., 2012; Zhang et al., 2017); Cotton’s study showed that optimal interventions need to be at least 2–3 h per week for approximately two years (Cotton, 2002); and Tzuriel’s study found that interventions of 2 h per week for one semester resulted in significant results (Tzuriel et al., 2021). The short duration of the assessment in this experiment may explain why the CDA group significantly improved their ability on cue, but were unable to maintain their advantage over the NDA group in the posttest.

The present study also revealed an important phenomenon: after the pretest, the posttest phase of the test took significantly less time in the NDA group compared to the pretest. Given that there was no statistically significant difference between the pre and posttest scores of the NDA group, we hypothesized that the decrease in test time may be due to some children mastering the rules of the questions. These children were able to recall and recognize known answers more quickly, but may have had difficulty correcting initially incorrect answers. These findings suggest that computerized dynamic assessment can be effective in improving the efficiency of response in children with autism (Resing et al., 2020b; Veerbeek & Vogelaar, 2023).

### 5.2. An Analysis of the Effect of Initial Ability Level on Graphical Analogical Reasoning in Children with Autism

The study showed a significant negative correlation between actual and gain scores, i.e., the higher the actual scores, the lower the gain scores, indicating that learners with high actual scores made less progress, while those with low actual scores made more progress, and vice versa. This implies that lower achieving children with autism benefit more from computerized dynamic assessment than higher achieving children. This result is consistent with several studies that children with cognitive deficits tend to benefit more from support compared to cognitively competent learners (Izadi et al., 2023; Jaeggi et al., 2008; Luwel et al., 2011; Poehner & Lantolf, 2013; Swanson & Lussier, 2001; Tzuriel et al., 2021). Overall, this finding reveals a huge untapped potential for children with low cognitive ability and highlights the importance of developing this type of learner. However, this does not mean that children with rich prior knowledge should be ignored; after all, they usually perform better on complex tasks (Stevenson et al., 2013b). Their rich knowledge base provides them with more thinking material and problem-solving methods, enabling them to analyze and process complex information more efficiently. For this group of children, they also have their own unique developmental needs. Appropriate challenges and extended learning content are essential to stimulate their creativity and innovative thinking. Neglecting their needs may result in their potential not being fully realized and may even cause them to suffer from learning burnout and lack of motivation. Therefore, in assessment, we need to construct a multi-level and diversified system.

In addition, it was found that there is a complex correlation between time spent on answering questions and scores for children of different ability levels. In the intervention phase, the CDA group showed a significant negative correlation between time spent answering questions and test scores, which implies that children with higher ability levels spent less time during the intervention process. An interesting phenomenon was observed in the NDA group: when children scored less than 19.58, i.e., at lower ability levels, time spent answering questions was positively correlated with their scores; whereas, for those who scored more than 19.58 and had a higher level of ability, the scores were negatively correlated with the time spent, which indicates that they spent less time answering the questions. They took less time to answer the questions. This suggests that the cognitive ability of children in the low-scoring group may still be developing, and that they need more time to understand the questions, retrieve knowledge, and organize their thoughts to answer the questions correctly and obtain high scores after thorough consideration. These children are not incompetent, they just need more time to think and solve problems (Goldhammer et al., 2017, 2014). Children with high scores may have a solid knowledge base and a mature thinking mode, and can quickly grasp the key to the question, quickly call upon their knowledge to answer the question, and respond quickly and correctly, reflecting a high level of information processing and problem-solving ability.

Even for CDA—non-social children with the same level of a priori knowledge, the provision of prompts resulted in different levels of learning potential. Although the subjects have similar educational backgrounds and consistent learning progress, there are still differences in each individual’s cognitive strategies or learning style (Chuang et al., 2021). Different children have different types of needs for prompts. Some children only need metacognitive-level prompts, while others require more comprehensive and specific explanations (Resing et al., 2012). Additionally, different children prefer different ways of presenting prompts. Research has shown that different types of intervention methods, such as text-based and visual ones, have different impacts on children with ASD (Omar & Bidin, 2015). And different subjects and study materials may also lead to changes in learners’ prompt preferences (Oxford, 2016; Reid, 1987). This study infers that different children with ASD have different preferences for prompt types, which affect the learning outcomes. Consequently, even if they have the same prior knowledge level (the same pre-test scores), the potential scores they can achieve are different. This highlights the strengths of CDA as it can effectively identify and assess the developmental potential of children with autism. This advantage lays the foundation for subsequent individualized interventions for children with autism. The results of Rubble’s study suggest that individualized interventions for children with autism achieve better results and produce greater effect sizes (Ruble et al., 2010). Bakhoda conducted experiments with the general population, whereby different types of prompts, such as image and textual prompts, were provided according to the participants’ requirements. The study showed that this method of selecting individualized prompts as advocated by computerized dynamic assessment was effective in improving English reading comprehension (Bakhoda & Shabani, 2019). Therefore, during the assessment of children with autism and subsequent interventions, educators should first consider the initial proficiency level of the learner and implement targeted instruments and measures to promote more effective progress for children with autism (Zhang et al., 2017).

## 6. Limitations

The limitations of this study lie in the fact that the implementation of CDA must rely on a digital environment. This approach may inadvertently exclude individuals on the autism spectrum with sensory hypersensitivity (e.g., aversion to screen brightness, specific auditory stimuli, or touch-screen interactions). Such potential barriers may affect the representativeness of the sample, and thus the study results may primarily apply to autistic individuals with a higher affinity for digital technologies. In future research, it is recommended to develop a CDA platform with customizable sensory parameters (e.g., adjustable screen chromaticity, audio frequency range, and tactile feedback intensity); implement a hybrid education model that integrates digital tasks with tangible alternatives to accommodate differences in sensory sensitivity.

## 7. Conclusions and Implications

This study delved into the effects of computerized dynamic assessment on the graphical analogical reasoning ability of children with autism. The results show that computerized dynamic assessment is effective in stimulating the analogical reasoning potential of children with autism, but the effect is short-lived and has not yet developed a sustained impact. At the same initial ability level, the presence or absence of a prompt caused children to show different learning potentials; children with lower initial levels benefited more from computerized dynamic assessment. At the same time, there were differences in the cognitive patterns of children at different initial levels, with children at higher initial levels recognizing graphical information more quickly and accurately and achieving better results, while children at lower initial levels needed more effort to improve their graphical analogical reasoning. Overall, these results help to accurately identify individual differences in children with autism and provide a basis for individualized education.

In future educational practices, whether it is cognitive learning or non-cognitive learning such as social skills and emotion recognition, CDA can be adopted. When selecting mediators, in addition to teachers, interesting devices such as robots can also be chosen to enhance the learning interest of children with autism (Resing et al., 2020b). When designing the prompt content, it is recommended that designers first prepare a basic version. During subsequent one-on-one personalized training, personalized plans can be developed according to the children’s preferences and needs (Naeini & Duvall, 2012). In addition, considering the cognitive peculiarities of children with autism, they may need more levels of prompts (Missiuna & Samuels, 1989). When presenting prompts, multimedia means such as audio and video can be used. Most learners prefer visual prompts; for example in scenarios such as social interactions, VR technology can be used for auxiliary presentation (Bakhoda & Shabani, 2019). In terms of intervention time, an intervention plan with a semester as the unit can be formulated, since significant learning outcomes for children require a relatively long-term intervention. In conclusion, CDA has broad application prospects and important practical value in the education of children with autism. Reasonable design can help them achieve better development in cognitive and non-cognitive fields, gradually integrate into society, and embark on a better future.

## Figures and Tables

**Table 1:** Basic demographic information.

Basic Information	CDA (*n* = 34)	NDA (*n* = 32)	z	*p*
Sex (M:F)	27:7	24:7	−1.897	0.058
Age (M ± SD)	6.07 ± 1.44	5.49 ± 1.29		

Abbreviations: F, Female; M, Male.

**Table 2:** Graphic transformation rules.

Transformation Rules		Specific Transformation
Quantity		Increase in the number of elements
Size		Elemental area becomes larger
Color		Blue element becomes green element
Shape		Half of an element disappears
Position	Nesting	Left element is nested within right element
	Overlapping	Two separated elements are placed on top of each other
Conversion	Inversion	Element is flipped vertically
	Rotation	Element rotated 90 degrees

**Table 3:** Three-Level Prompt Design.

Prompt Level	Illustrations	Prompt	Point
Level 1	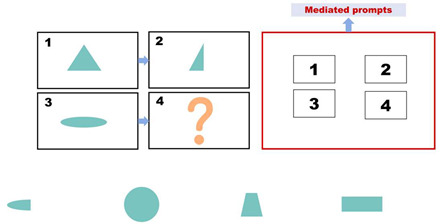	Children, please focus your attention and carefully consider the pattern of changes from Figure 1 to Figure 2. Use this pattern to infer the changes from Figure 3 to Figure 4, and subsequently determine the shape of Figure 4. Please indicate your answer using your hand.	3
Level 2	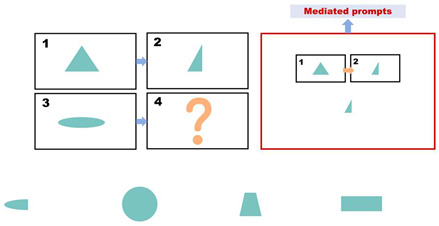	The transition from Figure 1 to Figure 2 involves a shape change, where the right half of the triangle is removed. Similarly, the transition from Figure 3 to Figure 4 follows the same shape change. Please indicate your answer using your hand.	2
Level 3	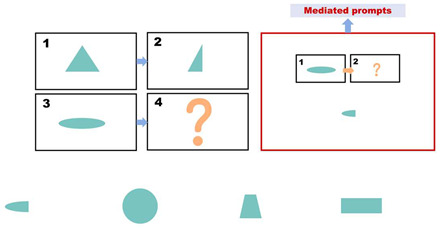	The correct choice would be the first option, where the right half of the oval is removed.	1

**Table 4:** The results of the GEE analysis for test scores.

Group	Pretest	Intervention	Posttest	$\chi^{2}$	*p*
CDA (n = 36)	20.00 (11.00, 28.00)	26.50 (16.50, 31.00)	20.00 (8.00, 28.00)	16.467	<0.001
NDA (n = 35)	12.00 (8.00, 26.00)	12.00 (8.00, 27.00)	14.00 (5.00, 24.00)	5.614	0.060
z	−1.213	−3.214	−1.900		
*p*	0.225	0.001	0.057		
$\mathrm{Wald}\;\chi^{2}$$\;\mathrm{Group}\;=\;5.539;\;\mathrm{Wald}\;\chi^{2}$$\;\mathrm{Phase}\;=\;30.886;\;\mathrm{Wald}\;\chi^{2}$ Interaction = 15.711					
*p* Group = 0.019; *p* Phase < 0.001; *p* Interaction < 0.001					

**Table 5:** The results of the GEE analysis for time spent.

Group	Pretest	Posttest	z	*p*
CDA (n = 27)	63.00 (41.50, 101.25)	45.00 (24.00, 77.25)	−2.078	0.038
NDA (n = 20)	65.00 (45.50, 115.00)	49.50 (32.25, 81.75)	−3.498	<0.001
z	0.160	0.154		
*p*	0.873	0.878		
$\mathrm{Wald}\;\chi^{2}$$\;\mathrm{Group}\;=\;0.887;\;\mathrm{Wald}\;\chi^{2}$$\;\mathrm{Phase}\;=\;0.032;\;\mathrm{Wald}\;\chi^{2}$ Interaction = 0.961				
*p* Group = 0.346; *p* Phase = 0.858; *p* Interaction = 0.327				

**Table 6:** Results of the GEE analysis of fixation count.

Group	Pretest	Intervention	Posttest	$\chi^{2}$	*p*
CDA (n = 20)	4.57 (3.00, 10.32)	5.91 (3.91, 11.58)	4.35 (2.47, 7.91)	2.154	0.341
NDA (n = 16)	6.34 (2.77, 8.09)	3.72 (2.92, 5.72)	3.36 (2.11, 5.17)	0.651	0.722
z	−0.139	−2.116	−1.096		
*p*	0.890	0.034	0.273		
$\mathrm{Wald}\;\chi^{2}$$\;\mathrm{Group}\;=\;3.454;\;\mathrm{Wald}\;\chi^{2}$$\;\mathrm{Phase}\;=\;6.938;\;\mathrm{Wald}\;\chi^{2}$ Interaction = 3.180					
*p* Group = 0.063; *p* Phase = 0.031; *p* Interaction = 0.204					

**Table 7:** Results of the GEE analysis of fixation duration mean.

Group	Pretest	Intervention	Posttest	$\chi^{2}$	*p*
CDA (n = 20)	272.62 (241.94, 326.52)	279.57 (252.69, 314.64)	279.20 (224.22, 318.27)	0.316	0.854
NDA (n = 16)	273.12 (227.35, 322.17)	277.06 (238.26, 298.68)	267.56 (225.51, 346.5025)	1.000	0.607
z	−0.024	−0.587	−0.147		
*p*	0.980	0.557	0.883		
$\mathrm{Wald}\;\chi^{2}$$\;\mathrm{Group}\;=\;0.954;\;\mathrm{Wald}\;\chi^{2}$$\;\mathrm{Phase}\;=\;2.694;\;\mathrm{Wald}\;\chi^{2}$ Interaction = 1.448					
*p* Group = 0.329; *p* Phase = 0.260; *p* Interaction = 0.485					

**Table 8:** Hotspot map of children’s attention to experimental materials in the two groups at different stages of the experiment.

	Pretest	Intervention	Posttest
CDA	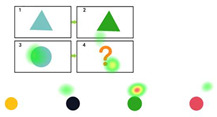	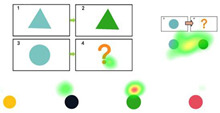	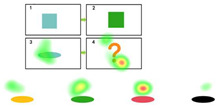
NDA	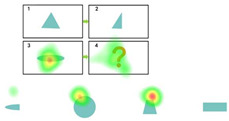	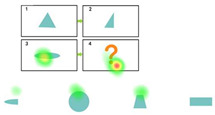	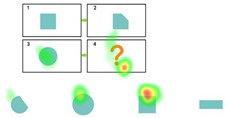

## Data Availability

The experimental data generated during the study are not publicly available due to privacy and ethical restrictions.

## Abbreviations

The following abbreviations are used in this manuscript:

ASD	Autism spectrum disorder
TD	Typically developing
DA	dynamic assessment
CDA	Computerized dynamic assessment
NDA	Non dynamic assessment
SCT	Sociocultural theory
EF	Executive function
ZPD	Zone of proximal development
